# Side-group-mediated thermoelectric properties of anthracene single-molecule junction with anchoring groups

**DOI:** 10.1038/s41598-021-88297-2

**Published:** 2021-04-26

**Authors:** Saeideh Ramezani Akbarabadi, Hamid Rahimpour Soleimani, Maysam Bagheri Tagani

**Affiliations:** Computational Nanophysics Laboratory (CNL), Department of Physics, University of Guilan, Rasht, 41335-1914 Iran

**Keywords:** Chemical physics, Condensed-matter physics

## Abstract

Charge transfer characteristics of single-molecule junctions at the nanoscale, and consequently, their thermoelectric properties can be dramatically tuned by chemical or conformational modification of side groups or anchoring groups. In this study, we used density functional theory (DFT) combined with the non-equilibrium Green’s function (NEGF) formalism in the linear response regime to examine the thermoelectric properties of a side-group-mediated anthracene molecule coupled to gold (Au) electrodes via anchoring groups. In order to provide a comparative inspection three different side groups, i.e. amine, nitro and methyl, in two different positions were considered for the functionalization of the molecule terminated with thiol or isocyanide anchoring groups. We showed that when the anchored molecule is perturbed with side group, the peaks of the transmission spectrum were shifted relative to the Fermi energy in comparison to the unperturbed molecule (i.e. without side group) leading to modified thermoelectric properties of the system. Particularly, in the thiol-terminated molecule the amine side group showed the greatest figure of merit in both positions which was suppressed by the change of side group position. However, in the isocyanide-terminated molecule the methyl side group attained the greatest thermoelectric efficiency where its magnitude was relatively robust to the change of side group position. In this way, different combinations of side groups and anchoring groups can improve or suppress thermopower and the figure of merit of the molecular junction depending on the interplay between charge donating/accepting nature of the functionals or their position.

## Introduction

High performance nanoscale molecular junctions where a single molecule is coupled two metal electrodes provide a unique opportunity to address future energy needs by employing thermoelectric effect which can be potentially used for a wide range of applications^1,2^. The efficiency of a thermoelectric device can be described by a dimensionless parameter termed as the figure of merit (*ZT*). The figure of merit depends on the thermopower or Seebeck coefficient (*S*), operating temperature (*T*), electrical conductance (*G*) and thermal conductance (*K*) which is defined as $ZT = S^2 T G / K$. The thermal conductance is composed of electron ($K_{\mathrm{el}}$) and phonon ($K_{\mathrm{ph}}$) contributions^3^, i.e. $K = K_{\mathrm{el}} + K_{\mathrm{ph}}$. Due to the interplay between several thermoelectric coefficients, obtaining a high value for the thermoelectric efficiency seems to be a challenging task suggesting the need for modified molecular structures with engineered properties^4–6^.

During the past two decades, the development of experimental techniques made it possible to measure the thermoelectric properties at the level of single molecules, providing new insights into different aspects of structure-function relationship in single-molecule junctions (recently reviewed by Wang et al.^7^). For instance, experimental techniques such as scanning tunneling microscope break junction (STM-BJ)^8–11^, mechanically controllable break junction (MCBJ)^12,13^ and electromigrated break junction (EMBJ)^14^ were developed to measure the thermopower of single-molecule junctions connected to metal electrodes—mostly made of gold (Au)—to form a metal-molecule-metal structure. Furthermore, conductive atomic force microscope (C-AFM) technique was used for thermopower measurements in networks of molecular junctions featuring multiple molecules^15,16^. The measurement of the thermopower in single-molecule junctions was first reported by Reddy et al. employing a STM-BJ method via switching between a standard STM current mode and voltage mode^11^. Later, by using a modified STM-BJ based technique, Widawsky et al. were able to simultaneously measure the conductance and thermopower of Au-linked single-molecule junctions via direct measurement of electrical and thermoelectric currents under zero external bias^8^. Most recently, Cui et al. used picowatt-resolution scanning probes to measure thermal conductance in single-molecule junctions^17^. Furthermore, by combining the break junction technique with suspended heat-flux sensors, Mosso et al. simultaneously measured the thermal and electrical conductances in organic single-molecule junctions with Au electrodes for the first time^18^.

The thermoelectric properties of single-molecule junctions can be modified by manipulating their transport properties which is extensively explored by theoretical and experimental studies. For example, increasing the length of the molecule^9,16,19–22^, changing the molecule-electrode coupling geometry^23–25^ or chemical doping of the molecule^6,26^ can substantially modify the conductance and thermopower in single-molecule junctions, depending on the system. More effectively, structural or chemical modification of anchoring groups^27–33^ can significantly tune the thermoelectric properties of molecular junctions. Particularly, theoretical studies showed that anchoring groups can control the electrical conductance^31,33^ or the thermal conductance^27^ in single-molecule junctions by the energy-level line up relative to the metal Fermi energy either by chemical engineering or conformational changes of the anchoring units. For example, thiol and isocyanide anchoring groups couple to the Au electrodes with different strengths and can breakdown the symmetry of the transmission peaks due to their electron-donating/accepting nature, respectively, resulting in an enhanced or suppressed conductance depending on the molecular structure^32,33^. The nature of anchoring group can also determine the sign of the thermopower of molecular junctions (which reveals the nature of the transport) by modifying energy separation between the highest occupied molecular orbital (HOMO) and the lowest unoccupied molecular orbital (LUMO), i.e. the ${\mathrm{HOMO-LUMO}}$ gap^30,34^. These theoretical findings were consistent with experimental measurements in single-molecule junctions with Au electrodes realized by STM-BJ^35^ or MCBJ^30^ techniques, implying that anchoring groups critically determine the charge transport direction through the system by the rearrangement of HOMO or LUMO level.

Functionalization of the molecule with side groups can also have dramatic effects on the thermoelectric properties of molecular junctions. Theoretical findings suggested that conformational or chemical modification of side groups tunes thermoelectric transport properties of molecular junctions by controlling charge polarization between the molecule and the electrode which is believed to be strongly correlated with the reorganization of the frontier molecular orbitals (FMOs) relative to the Fermi energy ($E_{\mathrm{F}}$) level of the electrodes^36–41^. In fact, side group position or its electron-donating/accepting character (e.g. amine vs. nitro) can lead to resonance effects near the Fermi energy that are responsible for current flow in the molecular device^39,41^. In this way, the appearance of side-group-induced Fano resonances, e.g., can lead to the reduction of the thermal conductance in polycyclic organic molecules connected to Au electrodes when amine is used as the side group^38^. Moreover, destructive phonon interference due to the presence of radical side groups can significantly decrease phonon contribution to the thermal conductance, leading to the enhancement of thermoelectric efficiency^36^. Qualitatively similar results were obtained in single-molecule junctions with Au electrodes realized, e.g., by STM-BJ experiments. For example, it was shown that the nitro side group can increase/decrease the electrical conductance based on the type of the central molecule in the junction^37^. In addition, STM-BJ experiments suggested that side groups can adjust the electrical conductance of molecular junctions by controlling contact configuration during mechanical modulation^42,43^.

According to these theoretical and experimental findings, the most significant effect of anchoring groups or side groups in the regulation of transport characteristics of metal-molecule-metal structures is the induced energy level alignment between FMOs and the Fermi level of electrodes^44,45^. Such a perturbation can be modulated, e.g., by the physical presence, position or charge donating/accepting character of anchoring groups^33,34,46^ or side groups^41,47^. The extent of the rearrangement of FMOs depends on the physical properties of the molecular device and the direction of charge transfer through the system^46^. In this way, appropriate choice of functional groups can modify the nature of transport in molecular junctions, i.e., p-type vs. n-type^41,46,48^. The possibility to manipulate the charge transfer direction in single-molecule junctions offers a unique opportunity to improve the thermoelectric efficiency in systems operating at the nanoscale.

Most of the previous studies considered the effects of side groups or anchoring groups on the thermoelectric properties of single-molecule junctions separately, i.e., the effect of (the chemical/conformational changes of) side groups in the presence of a single anchoring group or the effect of various (chemical/conformational settings of) anchoring groups in the absence/presence of a single side group. Here, however, we combined various configurations to further include the interplay between side groups and anchoring groups, i.e., we assumed three different side groups in two different positions when the molecule was attached to the Au electrodes with two different anchoring groups (comprising 12 distinct configurations) and further compared the results with the unperturbed molecule (i.e. without side groups). Particularly, we considered an organic molecule functionalized with side groups attached to two Au electrodes via anchoring groups and comparatively explored the influence of conformational changes and chemical nature of different side groups on the thermoelectric coefficients of the system in the presence of anchoring groups. We used anthracene as the central molecule that is a polycyclic aromatic hydrocarbon of formula ${\mathrm{C_{14}H_{10}}}$ composed of three fused benzene rings. Anthracene contains $\pi$-orbitals that contribute in the transport and can lead to the high conductance at the metal-molecule interface, especially when Au is used as the electrode^49^, which makes it a suitable candidate for thermoelectric effect studies. We used three different chemical compounds, i.e. amine ($- {\mathrm{NH_2}}$), nitro ($- {\mathrm{NO_2}}$) and methyl ($- {\mathrm{CH_3}}$), as the side group in two different geometric positions, i.e. ${\mathrm{R^{\prime }}}$ and ${\mathrm{R}}$ positions. Furthermore, the coupling of the molecule to the electrodes was mediated by two anchoring groups, i.e., thiol ($- {\mathrm{SH}}$) or isocyanide ($- {\mathrm{NC}}$).

We first ignored the role of side groups and inspected the behavior of transmission and thermoelectric coefficients only when the molecule is coupled to the Au electrodes via anchoring groups (i.e. unperturbed molecule). We then functionalized the molecule with three different side groups (i.e. perturbed molecule), each in two different positions, in the presence of two different anchoring groups and compared the behavior of electrical conductance, thermal conductance, thermopower and figure of merit in different configurations. In this way, the anthracene single-molecule junction can be modified by directly introducing side groups in two different positions where electron-rich properties and large $\pi$-conjugation system enhance the energy separation level of HOMO and LUMO. Our results show that in the perturbed molecule the peaks of transmission (with modified sharpness) are shifted relative to the Fermi energy in comparison to the unperturbed molecule. This chemical gating of the transmission spectrum can ultimately shape the behavior of thermoelectric coefficients. Particularly, these modifications determine the nature of transport and the sign of the thermopower leading to an enhanced or suppressed figure of merit depending on the charge transfer character and the position of side groups and anchoring groups.

Another aspect of our study is that since both the electrical conductance and electronic thermal conductance were calculated, we also quantitatively checked if the Wiedemann–Franz law holds in the Au-anthracene-Au single-molecule junction considered here (see Supplementary Information). Ultimately, in order to provide a comparative evaluation, the presented results for the thermoelectric coefficients at $T = 300 \, {\mathrm{K}}$ were summarized in Table 1 in a color-coded manner for easier inspection to better illustrate the interplay between various configurations. This comparison revealed that in the thiol-terminated molecule perturbed with side groups the trend of all thermoelectric coefficients for each position is preserved, i.e. amine > nitro > methyl in the ${\mathrm{R^{\prime }}}$ position and amine > methyl > nitro in the ${\mathrm{R}}$ position. In the isocyanide-terminated molecule, however, it seems that mostly the thermopower and figure of merit follow each other’s behavior leading to a relatively robust trend against the change of side group position, i.e. methyl > amine > nitro. Our results indicate that the interplay between the position/chemical nature of side groups and the chemical character of anchoring groups attached to the molecule is a key factor in the modification of thermoelectric properties of single-molecule junctions and indicate that a suitable choice of these functionals together can improve thermoelectric efficiency by the manipulation of charge transfer between the molecule and the electrodes.

## Methods

**Figure 1:**
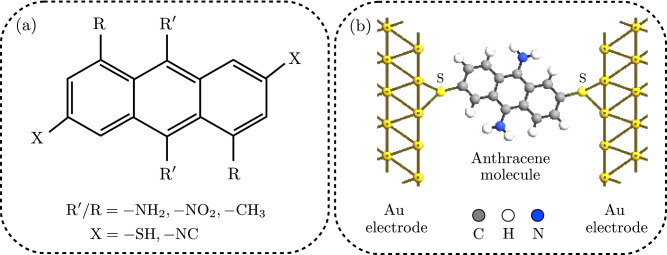
(**a**) Schematic representation of side groups in the $\mathrm{R^{\prime }}$ and $\mathrm{R}$ positions and anchoring groups in the $\mathrm{X}$ position attached to an anthracene molecule: $\mathrm{R}^{\prime }/\mathrm{R} =$ amine ($- \mathrm{NH_2}$), nitro ($- \mathrm{NO_2}$) or methyl ($- \mathrm{CH_3}$), and $\mathrm{X} =$ thiol ($- \mathrm{SH}$) or isocyanide ($- \mathrm{NC}$). (**b**) The examplary device model of the anthracene molecule with amine as the side group in the $\mathrm{R}^{\prime }$ position when the molecule is coupled to the hollow position of the tips of two Au (111) pyramids via the thiol anchoring group.

The geometric arrangement of side groups and anchoring groups attached to an anthracene molecule is representatively shown in Fig. 1a. We considered amine ($- {\mathrm{NH_2}}$), nitro ($-{{ \mathrm NO_2}}$) and methyl ($- {\mathrm{CH_3}}$) as three different side groups in two different positions labeled as ${\mathrm{R^{\prime }}}$ and ${\mathrm{R}}$ in Fig. 1a. Moreover, thiol ($- {\mathrm{SH}}$) or isocyanide ($- {\mathrm{NC}}$) anchoring groups in the $\mathrm{X}$ position (also labeled in Fig. 1a) were used to mediated the coupling of the anthracene molecule to the Au electrodes. One of the molecular structures considered in this study is examplary shown in Fig. 1b, where the anthracene molecule is attached to two three-dimensional Au electrodes via the thiol anchoring unit and is functionalized with the amine side group in the $\mathrm{R^{\prime }}$ position. Thiol and isocyanide anchoring groups were connected to the hollow and top positions fo the Au (111) electrodes, respectively, that is believed to be the most stable adsorption site for each anchoring unit^16,48^.

Density functional theory (DFT) based calculations were performed as implemented in the SIESTA computer program^50^. Transport properties were investigated by the non-equilibrium Green’s function (NEGF) formalism implemented in the TranSIESTA package^51^. The electronic structure calculations were carried out using DFT with the Perdew-Burke-Ernzerhof (PBE) generalized gradient approximation (GGA)^52^. We used $2 \times 2 \times 100$ k-point sampling in order to sample the Brillouin zone. The molecule was then extended to include surface layers of the Au leads. Three Au layers were considered as the left and right electrodes. The kinetic energy cutoff was chosen to be 75 Hartree.

Transport properties of the system were calculated by the Landauer-Buttiker transmission formalism expressed in terms of the Green’s function^53^:

1 $$\begin{aligned} T(\varepsilon ) = {\mathrm{Tr}} [{\mathbf {G}}^{{\mathrm {r}}}(\varepsilon ) \Gamma _{{\mathrm {L}}}(\varepsilon ) {\mathbf {G}}^{{\mathrm {a}}}(\varepsilon ) \Gamma _{{\mathrm {R}}}(\varepsilon )], \end{aligned}$$

where $\Gamma _{\alpha } = - 2 \, {\mathrm {Im}} \Sigma _{\alpha }$ is given in terms of the self-energy $\Sigma _{\alpha }$ of the lead $\alpha$, and ${ \mathbf {G}}^{{\mathrm {r}}}(\varepsilon )$ is the retarded Green’s function. The advanced Green’s function can be obtained by the relation ${ \mathbf {G}}^{{\mathrm {a}}}(\varepsilon ) = [ {\mathbf {G}}^{{\mathrm {r}}}(\varepsilon )]^{\dagger }$. Charge (*I*) and heat ($I_{\mathrm{Q}}$) currents calculations were performed by the Keldysh NEGF formalism^53^. In the linear response regime where the temperature gradient ($\Delta T$) and voltage difference ($\Delta V$) are small, the charge and heat currents flowing through the system can be expanded to the first order in terms of $\Delta T$ and $\Delta V$ as follows^53,54^:

2 $$\begin{aligned} \begin{array}{l} I = e^2 L_0 \Delta V + \dfrac{e}{T} L_1 \Delta T,\\ I_{\mathrm{Q}} = - e L_1 \Delta V - \dfrac{1}{T} L_2 \Delta T, \end{array} \end{aligned}$$

where $L_n = \hbar ^{-1} \int d \varepsilon (\varepsilon - \mu )^n \, T(\varepsilon ) \, (-\partial f(\varepsilon ) / \partial \varepsilon )$ is the Lorenz function, $f(\varepsilon )$ is the equilibrium Fermi-Dirac function, and $\mu$ is the chemical potential.

The thermoelectric coefficients are defined in terms of the $L_n$ quantities and can be determined by the transmission properties of the molecular system. For instance, the electrical conductance and the electron contribution to the thermal conductance can be expressed as follows, respectively:

3 $$\begin{aligned} G= & {} e^2 L_0, \end{aligned}$$

4 $$\begin{aligned} K_{\mathrm{el}}= & {} (1 / T) (L_2 - L_1^2 / L_0). \end{aligned}$$

The thermoelectric coefficients were calculated in the linear response regime where both bias voltage and temperature differences between the two leads tend to zero, i.e. $\mu _{\mathrm {L}} = \mu _{\mathrm {R}} = \mu$ and $T_{\mathrm {L}} = T_{\mathrm {R}} = T$. The thermopower can then be defined as the ratio of the induced voltage difference to the applied temperature gradient in the vanishing current, as follows:

5 $$\begin{aligned} S = - \dfrac{\Delta V}{\Delta T} = - (1 / eT)(L_1 / L_0), \end{aligned}$$

The interplay between the thermoelectric coefficients ultimately determines the thermoelectric figure of merit of the system by the relation $ZT = S^2 T G / (K_{\mathrm{el}} + K_{\mathrm{ph}})$. Typically, a significant fraction of the thermal conductance is due to the electronic contribution, and hence, the phononic thermal conductance is ignored^6^. Therefore, a constant value of $30 \, {\mathrm pW/K}$ was assumed for the phonon contribution to the thermal conductance to yield the net value of *ZT*.

## Results

### Anthracene molecule with anchoring groups

We first considered the anthracene molecule that is coupled to the Au electrodes via thiol and isocyanide anchoring groups in the $\mathrm{X}$ position (shown in Fig. 1a) and ignored the effect of side groups. A proper choice of anchoring groups can determine the nature of transport in the molecular junction to be either p-type (HOMO-dominated) or n-type (LUMO-dominated)^16,34,46,48^. This difference can be addressed by inspecting the electrode-coupling induced charge transfer ($\Delta N$) for each anchoring group^55^ that quantifies the change of the number of electrons in the isolated molecule (IM) and the extended molecule (EM) as $\Delta N = N_{\mathrm {IM}} - N_{\mathrm {EM}}$^34^. $N_{\mathrm {IM}}$ and $N_{\mathrm {EM}}$ are the number of electrons in the IM and EM systems, respectively. $N_{\mathrm {EM}}$ can be computed by defining the single particle density matrix ($\hat{\rho }$) in the natural atomic orbital basis and calculating its trace as $N_{\mathrm {EM}} = \mathrm{{Tr}} (\hat{\rho })$^34^. Calculated $\Delta N$ values for considered anchoring groups revealed that thiol molecule gains a 0.112 electron partial charge upon contact with the Au atoms. In contrast, the isocyanide molecule loses a 0.016 electron partial charge to the Au atoms.

**Figure 2:**
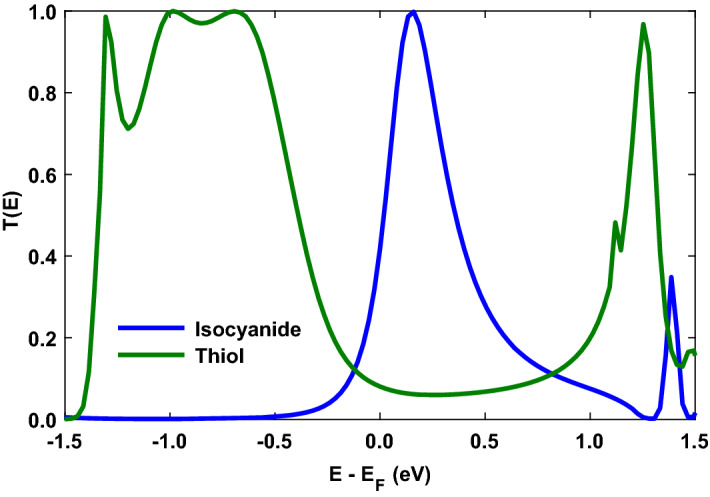
The transmission coefficient versus energy for the anthracene molecule coupled to the Au electrodes via thiol (green) and isocyanide (blue) anchoring groups evaluated at $T = 300 \, \mathrm{K}$.

This charge transfer analysis can give away charge donating/accepting character of the anchoring groups, and therefore, the nature of the transport by the rearrangement of FMOs^44^ when they mediate the coupling of the anthracene molecule to the Au electrodes^16^. The calculated charge transfer between the anthracene molecule and contacting Au atoms in the case of the thiol unit is $\Delta N_{\mathrm{SH}} = - 0.112$, representing charge (electron) transfer into the molecule which shifts the FMOs to higher energies. In contrast, the charge transfer for the isocyanide unit is $\Delta N_{\mathrm{NC}} = 0.016$, indicating a charge transfer out of the molecule that shifts the FMOs to lower energies. In this way, the thiol-terminated anthracene molecular device shows p-type (HOMO-dominated) transport, whereas the same device terminated with the isocyanide anchoring group shows n-type (LUMO-dominated) transport. This is shown in Fig. 2 where the transmission coefficient is depicted for the anthracene molecule coupled to the Au electrodes via thiol or isocyanide anchoring groups. In the case of the thiol anchoring group (green), the HOMO energy is located closer to the Fermi energy (p-type transport) since the electron-donating nature of the thiol unit increases the energy of $\pi$-electron system. In contrast, for the electron-accepting isocyanide anchoring group (blue), the electron transport occurs through the LUMO (n-type transport) which is in agreement with the results of previous studies^16,34,46^.

Moreover, the direction of charge transfer in the system can determined by the difference in the electronegativity ($\chi$) of the molecule and that of the Au electrodes, defined as the average of ionization energy and electron affinity. The electronegativity of the Au electrodes in the hollow site configuration (in contact model for the thiol-anchored molecule) is $\chi _{\mathrm{Au}}(\mathrm{hollow}) = 4.98 \, \mathrm{eV}$, whereas in the top site configuration (in contact model for the isocyanide-anchored molecule) is $\chi _{\mathrm{Au}}(\mathrm{top}) = 4.90 \, \mathrm{eV}$^46^. On the other hand, the electronegativity of the thiol unit is $\chi _{\mathrm{SH}} = 5.23 \, \mathrm{eV}$^46^ that is greater than that of the Au electrodes which explains the previously calculated charge transfer into the molecule (i.e. $\Delta N_{\mathrm{SH}} = - 0.112$), whereas the electronegativity of the isocyanide unit is $\chi _{\mathrm{NC}} = 4.39 \, \mathrm{eV}$^46^ that is less than the electronegativity of the Au electrodes implying charge transfer out of the molecule (i.e. $\Delta N_{\mathrm{NC}} = 0.016$). The interplay between charge polarization properties determined by the anchoring groups or the Au electrodes (and those of the side groups) can ultimately shape the behavior of the transmission coefficient (i.e. HOMO- or LUMO-dominated transport) in different configurations.

**Figure 3:**
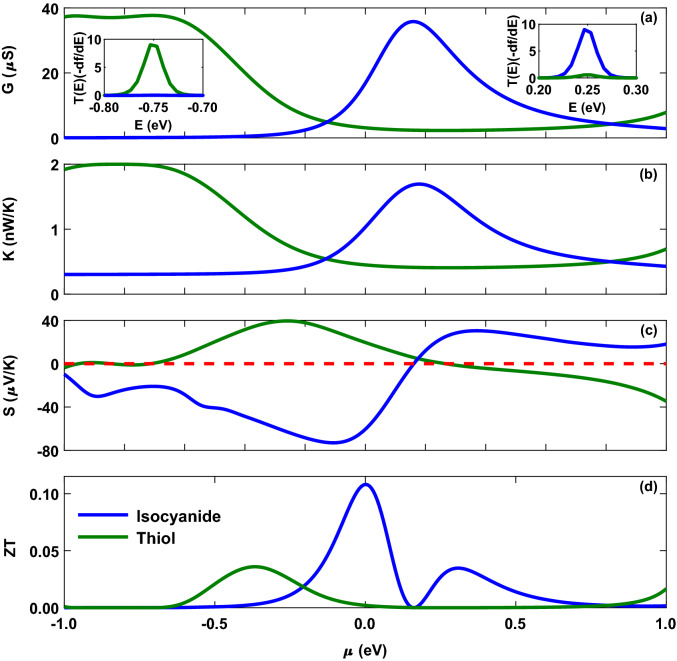
Thermoelectric coefficients of the Au-anthracene-Au junction as a function of the chemical potential of the electrodes with thiol (green) and isocyanide (blue) anchoring groups. (**a**) Electrical conductance, (**b**) thermal conductance, (**c**) thermopower (with $S = 0$ in red dashed line as a reference) and (**d**) figure of merit evaluated at $T = 300 \, \mathrm{K}$. Insets to panel (**a**) show $T(\varepsilon )(-\partial f/ \partial \varepsilon )$ as a function of energy.

The reorganization of the molecular energy levels and the dominant carriers participating in transport are strongly related to the change of chemical potential of the electrodes or an applied gate voltage^56^. To explore the effect of chemical potential of the electrodes on the thermoelectric properties of the molecular junction with thiol and isocyanide anchoring groups, the related thermoelectric coefficients are sketched in Fig. 3. The electrical conductance of the thiol-terminated molecular junction in Fig. 3a (green) is greater in negative chemical potentials due to the HOMO peak in the corresponding transmission coefficient in Fig. 2 (green) that is located closer to the Fermi energy. This can be further validated by the behavior of $T(\varepsilon )(-\partial f/ \partial \varepsilon )$ shown in the left inset to Fig. 3a. Conversely, the value of the electrical conductance for the molecular junction with isocyanide anchoring group increases in positive chemical potentials (see Fig. 3a, blue) in comparison with the thiol-terminated molecule that can be attributed to the LUMO peak in the transmission coefficient in Fig. 2 (blue) near the Fermi energy. The increase in the value of $T(\varepsilon )(-\partial f/ \partial \varepsilon )$ in the right inset to Fig. 3a verifies this behavior. The same arguments can be brought forward for the thermal conductance in Fig. 3b which qualitatively follows the behavior of the electrical conductance for both anchoring groups.

The thermopower of the molecular junction with thiol and isocyanide anchoring groups as a function of the chemical potential of the electrodes is shown in Fig. 3c where its oscillatory behavior originates from the change of the number of electrons contributing in transport. The sign of the thermopower can reveal information about the alignment of the HOMO and LUMO energies with respect to the Fermi energy ^57^. A positive thermopower indicates that HOMO lies closer to the Fermi energy than the LUMO implying a p-type transport, whereas a negative thermopower is obtained when LUMO is located closer to the Fermi energy which results in a n-type transport (cf. Figs. 2 and 3c). Moreover, the thermopower is zero at two points: Electron-hole symmetry points and resonance energies. In these energies, the sign of thermopower changes. In the symmetry points that result in zero net voltage, electrons and holes have equal contribution in transport but with opposite signs. The thermal conductance is maximum at these points since the Fermi derivative is broadened around the Fermi energy and more electrons and holes contribute in the conductance carrying more thermal energy in the same direction (see Fig. 3b). The thermopower also vanishes in the resonance energies since the temperature gradient cannot yield a net electrical current and electrons can tunnel from the electrodes to the molecular levels without requiring thermal energy^23^.

Taken together, Fig. 3c (green) shows that the thermopower in the thiol-terminated molecular junction is positive in negative chemical potentials and then decreases to negative values in positive chemical potentials due to the decrease in the transmission coefficient near the Fermi energy (also cf. green curves in the left and right insets to Fig. 3a). For the isocyanide-terminated device in Fig. 3c (blue), however, the situation is roughly reversed where the sign of the thermopower changes from negative to positive as the chemical potential varies from negative to positive values. This can be attributed to the location of the transmission coefficient peak near the Fermi energy in Fig. 2 (blue) which is further verified by the behavior of $T(\varepsilon )(-\partial f/ \partial \varepsilon )$ (also cf. blue curves in the left and right insets to Fig. 3a).

The behavior of the figure of merit strongly depends on the thermopower. The interplay between the electrical conductance, thermal conductance and thermopower ultimately shapes the figure of merit (i.e. $ZT = S^2 T G / K$) shown in Fig. 3d. For the molecular device with the thiol anchoring group (Fig. 3d, green), the maximum of the figure of merit occurs in negative chemical potentials due to a great contribution from the electrical conductance and thermopower in that range (see Fig. 3a and c, green). On the other hand, the value of the figure of merit increases in positive chemical potentials for the isocyanide anchoring group (Fig. 3d, blue) since in this case the molecular junction has greater electrical conductance and thermopower in this range (see Fig. 3a and c, blue). These observations indicate that how the chemical character of the anchoring group in a molecular junction can crucially impact on the thermoelectric coefficients, and particularly, the figure of merit.

**Figure 4:**
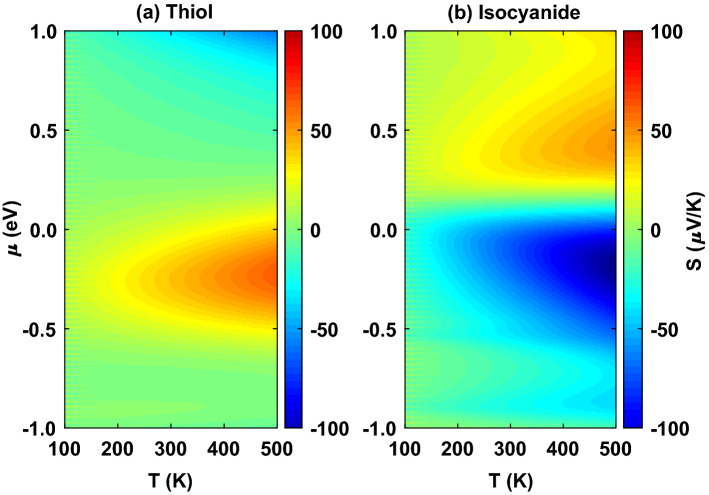
Temperature dependency of the thermopower when the chemical potential of the electrodes is varied systematically for the anthracene molecule coupled to the Au electrodes via (**a**) thiol and (**b**) isocyanide anchoring groups.

To take a look at the temperature dependency of the thermopower we systematically varied the chemical potential of the electrodes and calculated the thermopower as a function of temperature shown in Fig. 4. For both of the anchoring groups, the magnitude of thermopower is generally increased by temperature. In the case of the thiol anchoring group, the value of the thermopower is relatively increased with temperature (Fig. 4a) due to peaks in the transmission spectrum in Fig. 2 (green). However, the variation of thermopower is more pronounced when the molecule is mediated by the isocyanide anchoring group (Fig. 4b) due to an increase in the transmission coefficient around the Fermi energy (Fig. 2, blue) which increases $(\varepsilon - \mu )$ consequently. The temperature-dependent increase of the thermopower occurs in the vicinity of the Fermi energy since the transmission coefficient peak is located close to the Fermi energy and the overlap between the transmission coefficient and the Fermi derivative is notable in this range.

**Figure 5:**
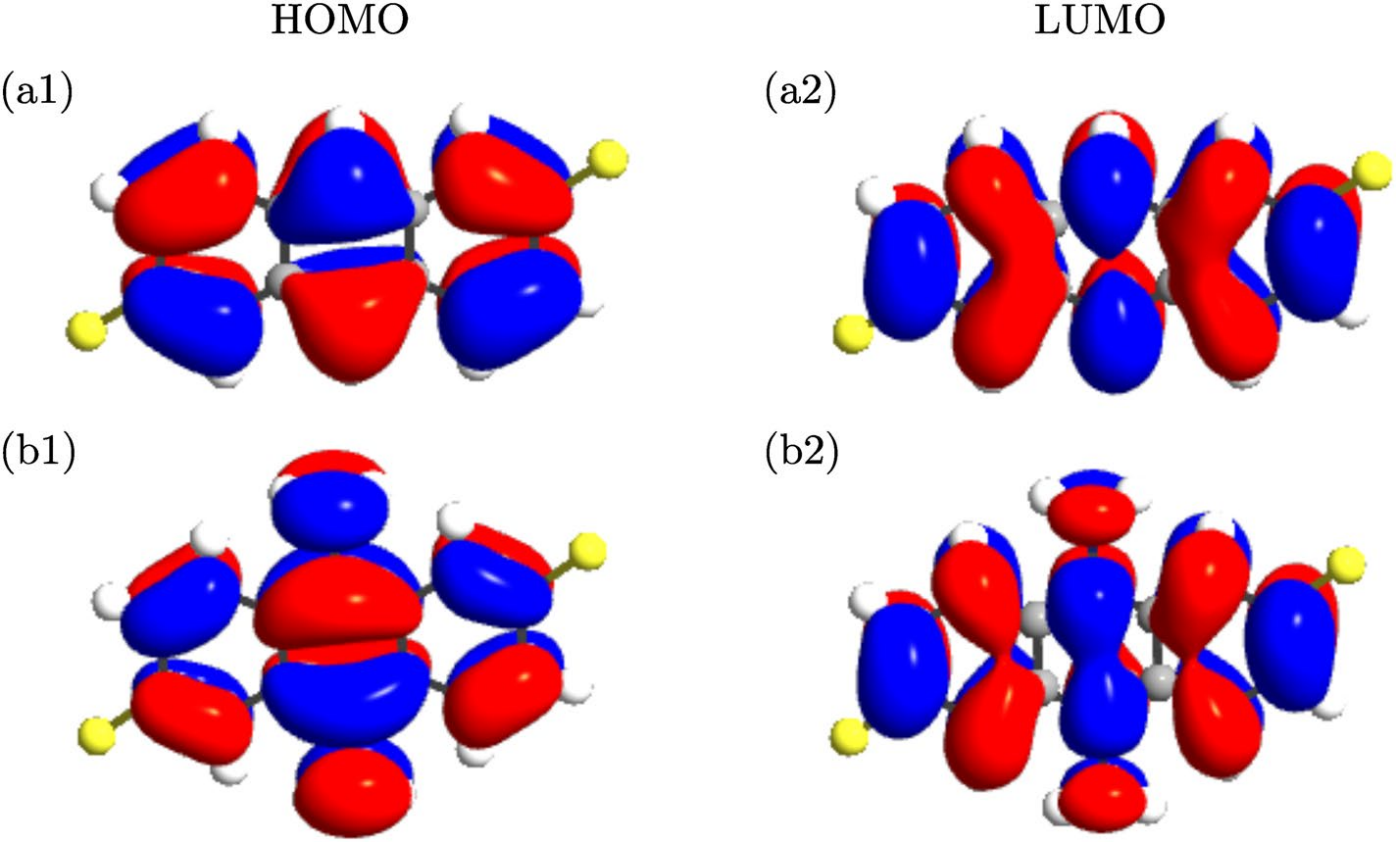
The calculated HOMO and LUMO orbitals of the anthracene molecule coupled to the thiol anchoring group, (**a1, a2**) in the absence of side groups, and (**b1, b2**) when the amine side group is attached to the molecule in the $\mathrm{R}^{\prime }$ position. The isovalue of each molecular orbital surface was 0.05 a.u.

### Side-group-mediated anthracene molecule with anchoring groups

So far, we only considered the role of two different anchoring groups on the thermoelectric properties of the Au-anthracene-Au molecular junction. Now we introduce three different side groups, i.e., amine ($- {\mathrm{NH}}_2$), nitro ($- {\mathrm{NO}}_2$) and methyl ($-{ \mathrm{CH}}_3$) in two different positions labeled as $\mathrm{R}^{\prime }$ and $\mathrm{R}$ in Fig. 1a where the molecule is attached to the Au electrodes via previously described thiol and isocyanide anchoring groups. Given the charge donating/accepting nature of each compound, different combination of side groups and anchoring groups may significantly modify thermoelectric properties of the molecular device.

**Figure 6:**
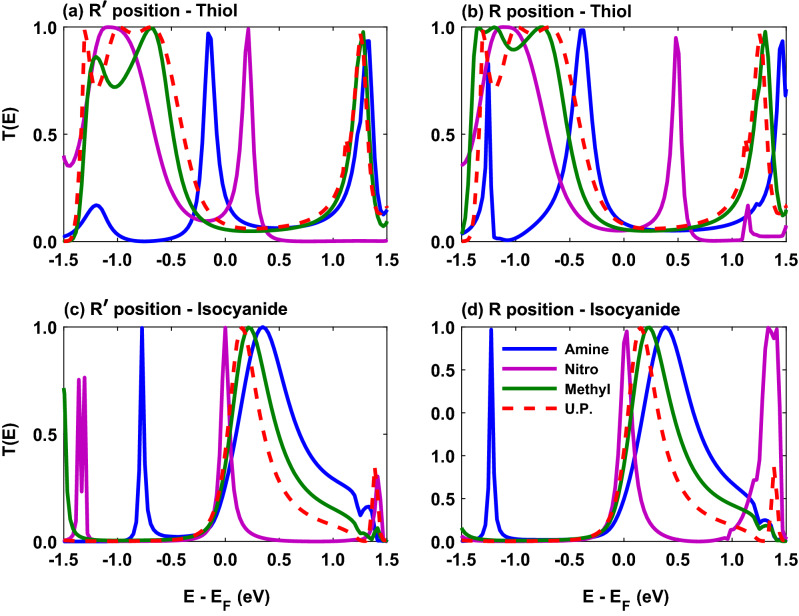
The transmission coefficient of the Au-anthracene-Au junction versus energy when the thiol anchoring group mediates molecule-electrode contact in the presence of amine (blue), nitro (magenta) or methyl (green) as the side group in (**a**) $\mathrm{R}^{\prime }$ and (**b**) $\mathrm{R}$ positions. The transmission coefficient of the isocyanide-terminated junction with amine (blue), nitro (magenta) or methyl (green) side group in (**c**) $\mathrm{R}^{\prime }$ and (**d**) $\mathrm{R}$ positions. Also, the transmission coefficient of the unperturbed (U.P.; red) system in the absence of side groups from Fig. 2 is redrawn to make the comparison easier.

The introduction of side groups can regulate HOMO or LUMO alignment, and consequently, the nature of transport depending on the donating/accepting character of the side group itself. To provide an example, the calculated HOMOs and LUMOs are shown in Fig. 5 when the unperturbed molecule (without side groups) is attached to the electrodes via the thiol anchoring group (a1, a2). The same system with the amine side group (i.e. perturbed molecule) is shown in Fig. 5b1 and b2. The states are anti-symmetric in the plane of the anthracene molecule implying that they represent the $\pi$-orbitals of the anthracene molecule, i.e., linear combination of the $\pi$-orbitals on each carbon atom perpendicular to the plane of the molecule. As shown in Fig. 5b1 and b2, HOMO is localized on the donor anthracene moiety due to the electron-donating nature of the amine side group. On the contrary, in the case of an electron-accepting side group (such as nitro; not shown) LUMO is localized over the acceptor unit due to the non-bonding lone pair of the N atom^58^. This difference can be ascribed to the additional side chain that is attached to the anthracene (e.g. in the ${\mathrm{R}}^{\prime }$ position in Fig. 5b1 and b2) which increases steric hindrance between the backbone and the amine side chain^39^. Therefore, in the case of molecular junction with the amine side group the HOMO energy is lowered as the electron-donating properties of the donor segment increase that is expected to enhance conduction in this case which can be validated by the transmission spectrum shown in Fig. 6a (blue). This effect is seen in push-pull systems where the HOMO energy is determined by the donor strength^59^.

The transmission coefficient of the molecular junction with amine, nitro and methyl side groups is shown in Fig. 6 when the connection of the molecule to the electrodes is mediated by thiol and isocyanide anchoring groups. A visual comparison between Fig. 6 and Fig. 2 (also shown by red dashed curve in Fig. 6) indicates that the introduction of side groups can shift the location of the transmission peaks or qualitatively modulate their shape. Moreover, the overall effect of the ${\mathrm{R}}^{\prime }$ and $\mathrm{R}$ positions of side groups can be viewed mainly as shifting the position of the transmission peaks. These phase shifts of transmission channels modify conduction in the molecular junction by the rearrangement of HOMO or LUMO energy levels with respect to the Fermi energy due to the quantum interference effects emerged among electronic waves passing through the molecular rings^60–62^. These modifications are a direct consequence of the position or nature of side groups attached to the molecule^41,63,64^.

**Figure 7:**
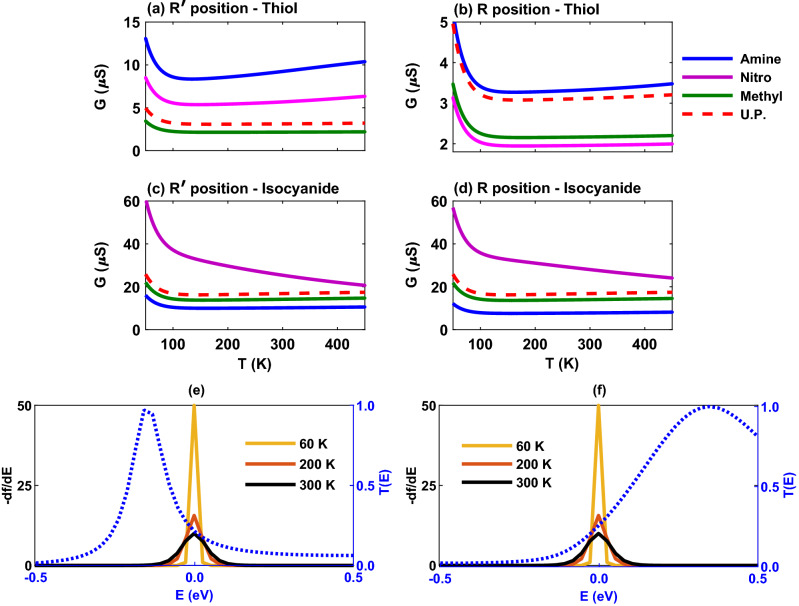
The electrical conductance of the Au-anthracene-Au junction as a function of temperature when the thiol anchoring group mediates molecule-electrode contact in the presence of amine (blue), nitro (magenta) or methyl (green) as the side group in (**a**) $\mathrm{R}^{\prime }$ and (**b**) $\mathrm{R}$ positions. The electrical conductance of the isocyanide-terminated junction with amine (blue), nitro (magenta) or methyl (green) side group in (**c**) ${\mathrm{R}}^{\prime }$ and (**d**) $\mathrm{R}$ positions. Moreover, the electrical conductance of the unperturbed (U.P.; red) system in the absence of side groups is drawn as a reference. Examplar representation of the Fermi derivative peak ($-df/d\varepsilon$ in the left axis; $T = 60,200,300 \, \mathrm{K}$) and transmission coefficient peak ($T(\varepsilon )$ in the right axis; blue dotted curve) for the molecule anchored with (**e**) thiol and (**f**) isocyanide units with amine side group in the $\mathrm{R}^{\prime }$ position.

The transmission coefficient in the thiol-anchored molecule is increased around the Fermi energy (with relatively sharp peaks) when amine (blue) and nitro (magenta) side groups are added to the system in the $\mathrm{R}^{\prime }$ position, whereas it remains relatively unaffected in the case of the methyl (green) side group in Fig. 6a in comparison to the unperturbed molecule (Fig. 6a, red curve). In the $\mathrm{R}$ position in Fig. 6b, HOMO (in amine) and LUMO (in nitro) energy levels are shifted away from the Fermi energy in comparison to the $\mathrm{R}^{\prime }$ position in a way that the transmission near the Fermi energy is suppressed and the $\mathrm{HOMO-LUMO}$ gap is increased. The molecule with the methyl side group, however, is relatively robust to this conformational change. Furthermore, Fig. 6 shows that generally for all side groups the $\mathrm{HOMO-LUMO}$ gap in the $\mathrm{R}$ position (panels b and d) is increased in comparison to the $\mathrm{R}^{\prime }$ position (panels a and c) in the case of both anchoring groups such that the transmission coefficient is reduced near the Fermi energy in the thiol-terminated molecule (Fig. 6b) whereas it is relatively unchanged in the isocyanide-terminated molecule (Fig. 6d). Particularly, when the molecule is anchored with isocyanide, the modulation of transmission due to the presence of side groups in the $\mathrm{R}^{\prime }$ position is more pronounced for the nitro (magenta) that resulted in a sharp peak at the Fermi energy in Fig. 6c in comparison to the transmission of the unperturbed molecule (Fig. 6c, red curve), whereas the moderately shifted transmission peaks for amine (blue) and methyl (green) side groups roughly preserved their shape near the Fermi energy. In this case, the effect of the $\mathrm{R}^{\prime }$ position of side groups is to slightly shift the transmission peaks towards higher energies in Fig. 6d, and therefore, increasing the $\mathrm{HOMO-LUMO}$ gap.

These different behaviors of the transmission coefficient in the presence of side groups in different positions can be ascribed to quantum interference effects^41,63,64^ and the interplay between charge donating and accepting characters of side groups and anchoring groups^33,39,65^. In fact, in Fig. 6a and b the transmission coefficient in the thiol-terminate molecule for the amine side group near the Fermi energy is more than other units since the amine side group is strongly electron-donating that is in accordance with the electron-donating nature of the thiol anchoring group. The methyl side group is also an electron-donating unit but weaker than the amine, and hence, its presence did not significantly affect the transmission coefficient. On the other hand, nitro is an electron-accepting side group which showed a LUMO-dominated transmission. However, when the molecule is anchored with isocyanide in Fig. 6c and d, the transmission coefficient around the Fermi energy is greater for the electron-accepting nitro unit (magenta) in both positions since its nature is similar to the electron-accepting character of the anchoring group, whereas the electron-donating amine (blue) and methyl (green) side groups has minor effect on the transmission in both positions in comparison to the molecule without side group (Fig. 6c and d, red curve). In addition, increased $\mathrm{HOMO-LUMO}$ gap in the $\mathrm{R}$ position with respect to the $\mathrm{R}^{\prime }$ position can be interpreted as a form of destructive quantum interference due to the side group position leading to a reduction in the conduction.

We then explored the behavior of the thermoelectric coefficients in the presence of amine, nitro and methyl side groups in two different positions when the molecule is anchored with thiol or isocyanide. The temperature dependency of the electrical conductance of the molecular junction is shown in Fig. 7a–d. For all of the side groups in both $\mathrm{R}^{\prime }$ and $\mathrm{R}$ positions, the electrical conductance decreases by temperature up to $T \sim 100 \, \mathrm{K}$ (see Fig. 7a–d). This descending trend of the electrical conductance is due to the behavior of the transmission coefficient and the broadening of the Fermi derivative ($-\partial f / \partial \varepsilon$) around the Fermi energy (shown in Fig. 7e) associated with $L_0$ in the electrical conductance relation given by Eq. (3) where its overlap with the peak of the transmission coefficient is decreased with temperature^6^. This ultimately decreases the number of carriers participating in transport resulting in a smaller electrical conductance. However, at higher temperatures the Fermi derivative gets wider and may cover additional peaks of the transmission coefficient. In such a case, the number of carriers participating in transport is increased leading to a greater electrical conductance, e.g., in case of the thiol-terminated molecule with amine side group (blue) in Fig. 7a. On the contrary, this may not happen as it is the case for the isocyanide-terminated molecule with nitro side group (magenta) in Fig. 7c.

For the thiol-terminated molecule, the greatest value of the electrical conductance corresponds to the amine side group in both $\mathrm{R}^{\prime }$ and $\mathrm{R}$ positions (cf. Fig. 7a and b). This was expected, since both the amine side group and the thiol anchoring groups share similar charge transfer properties, and furthermore, the transmission HOMO peak in the presence of the amine side group is located closer to the Fermi energy in comparison to other side groups (see Fig. 6a, blue). The same (but reversed) argument can be brought forward for the nitro side group but in this case the electrical conductance is reduced (Fig. 7a and b, magenta) due to the electron-accepting nature of nitro leading to a suppressed conduction. The lowest value of the electrical conductance in the $\mathrm{R}^{\prime }$ position corresponds to the methyl side group (Fig. 7a, green) due to the small transmission near the Fermi energy and a large $\mathrm{HOMO-LUMO}$ gap in the transmission spectrum (see Fig. 6a, green). A comparison between $\mathrm{R}^{\prime }$ and $\mathrm{R}$ positions reveals that the electrical conductance is reduced by changing the position of the side group from $\mathrm{R}^{\prime }$ to $\mathrm{R}$ for amine and nitro side groups, but the electrical conductance of the methyl side group is relatively robust to this conformational change (cf. Fig. 7a and b). The origin of this reduction comes from the observation that the $\mathrm{R}$ position increased the $\mathrm{HOMO-LUMO}$ gap in the transmission spectrum of amine and nitro side groups in comparison to the $\mathrm{R}^{\prime }$ position, except for the methyl unit that remained fairly unaffected.

Figure 7c and b shows the electrical conductance of the isocyanide-terminated molecule with side groups as a function of temperature. In this case, the lowest value of the electrical conductance belongs to the molecule with amine side group in both $\mathrm{R}^{\prime }$ and $\mathrm{R}$ positions (Fig. 7c and d, blue). This is due to the shape of the transmission peak of the molecule with amine side group near the Fermi energy (see Fig. 6c, blue) and its overlap with the Fermi derivative peak that is reduced by increasing temperature (Fig. 7f). The maximum value of the electrical conductance, however, corresponds to the molecule with nitro side group in both $\mathrm{R}^{\prime }$ and $\mathrm{R}$ positions (Fig. 7c and d, magenta) since both the side and anchoring units are electron-accepting and the peak of the transmission coefficient of the nitro-perturbed molecule is located extremely close to the Fermi energy leading to an enhanced conduction in this case (see Fig. 6c and d, magenta). The reduction of the electrical conductance with temperature in this case can be ascribed to the broadening of the Fermi derivative around the Fermi energy with temperature increasing (not shown) leading to a reduction of the number of carriers participating in transport that ultimately decreases the electrical conductance. By changing the position of side groups from $\mathrm{R}^{\prime }$ to $\mathrm{R}$, the electrical conductance for the amine-perturbed molecule is slightly decreased, whereas it is slightly increased in the case of nitro side group (cf. Fig. 7c and d). The electrical conductance of the molecule with methyl side group is relatively unaffected by this conformational change (cf. Fig. 7c and d, green). These observations are due to the behavior of $\mathrm{HOMO-LUMO}$ gap in the corresponding transmission spectrum of the molecule (see Fig. 6c and d).

**Figure 8:**
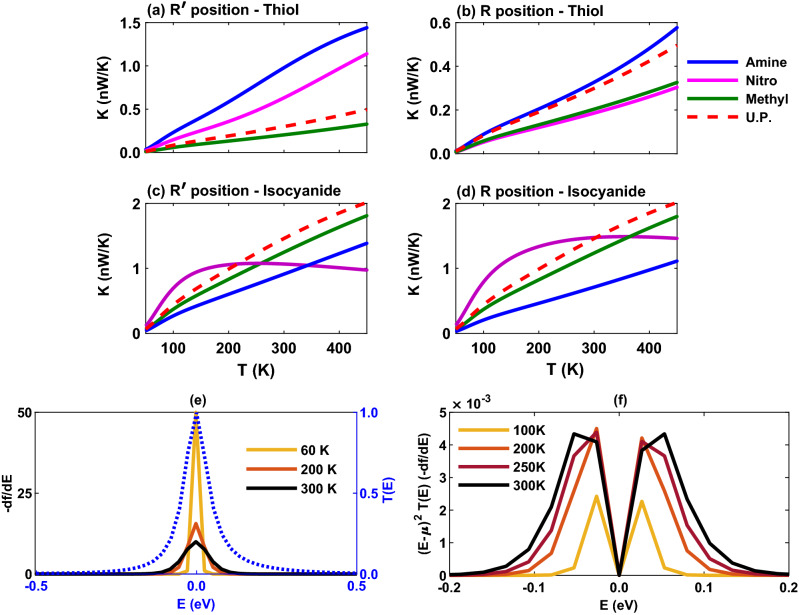
The thermal conductance of the Au-anthracene-Au junction as a function of temperature for the thiol-terminated junction in the presence of amine (blue), nitro (magenta) or methyl (green) as the side group in (**a**) $\mathrm{R}^{\prime }$ and (**b**) $\mathrm{R}$ positions. The electrical conductance of the isocyanide-terminated junction with amine (blue), nitro (magenta) or methyl (green) side group in (**c**) $\mathrm{R}^{\prime }$ and (**d**) $\mathrm{R}$ positions. Also, the thermal conductance of the unperturbed (U.P.; red) system in the absence of side groups is drawn as a reference. (**e**) Examplar representation of the Fermi derivative peak ($-df/d\varepsilon$ in the left axis; $T = 60,200,300 \, \mathrm{K}$) and transmission coefficient peak ($T(\varepsilon )$ in the right axis; blue dotted curve) for the molecule anchored with the isocyanide anchoring group with nitro side group in the $\mathrm{R}^{\prime }$ position. (**f**) $(\varepsilon -\mu )^2 T(\varepsilon )(-df/d\varepsilon )$ is drawn for the isocyanide-terminated junction with nitro side group in the $\mathrm{R}^{\prime }$ position.

The temperature dependency of the thermal conductance of the molecular junction is shown in Fig. 8a–d. Except the isocyanide-terminated molecule with nitro side group (Fig. 8c and d, magenta), the thermal conductance of the molecule is monotonically increased with temperature for all side groups in both $\mathrm{R}^{\prime }$ and $\mathrm{R}$ positions because electrons and holes carry more thermal energy when the temperature increases. In particular, in the thiol-terminated molecular junction the maximum value of the thermal conductance belongs to the molecule with amine side group in both $\mathrm{R}^{\prime }$ and $\mathrm{R}$ positions (Fig. 8a and b, blue). This can be attributed to the increased overlap between the transmission coefficient peak and the Fermi derivative peak and the contribution of $(\varepsilon -\mu )^2 T(\varepsilon )(-df/d\varepsilon )$ term (not shown) associated with $L_2$ in the thermal conductance expression given by Eq. (4) which increases with temperature. Furthermore, changing the position of side groups from $\mathrm{R}^{\prime }$ to $\mathrm R$ leads to the reduction of the thermal conductance due to the increased $\mathrm{HOMO-LUMO}$ gap in the transmission spectrum in the $\mathrm{R}$ position.

The thermal conductance of the isocyanide-terminated molecular junction is shown in Fig. 8c and d where it is increased with temperature in the case of amine (blue) and methyl (green) side groups in both $\mathrm{R}^{\prime }$ and $\mathrm{R}$ positions. However, for the molecule with the nitro side group the thermal conductance first increases with temperature up to $T \sim 200 \, \mathrm{K}$ in both positions (Fig. 8c and d, magenta) and then decreases. This behavior is due to the overlap between the transmission coefficient peak and the Fermi derivative peak that is increased up to $T \sim 200 \, \mathrm{K}$ and then decreased as shown in Fig. 8e. Furthermore, Fig. 8f shows that $(\varepsilon -\mu )^2 T(\varepsilon )(-df/d\varepsilon )$ (which provides the main contribution to the thermal conductance^6^) is increased near the Fermi energy up to $T \sim 200 \, \mathrm{K}$ (green) and then suppressed at higher temperatures. This explains the ascending and descending behavior of the thermal conductance in the molecule functionalized with the nitro side group. A comparison between $\mathrm{R}^{\prime }$ and $\mathrm{R}$ positions indicates that the value of thermal conductance in the isocyanide-terminated molecule is relatively preserved (amine and methyl) or slightly decreased (nitro) as opposed to the thiol-terminated junction where the thermal conductance is almost reduced to half (amine and nitro) or remained to an extent unchanged (methyl).

**Figure 9:**
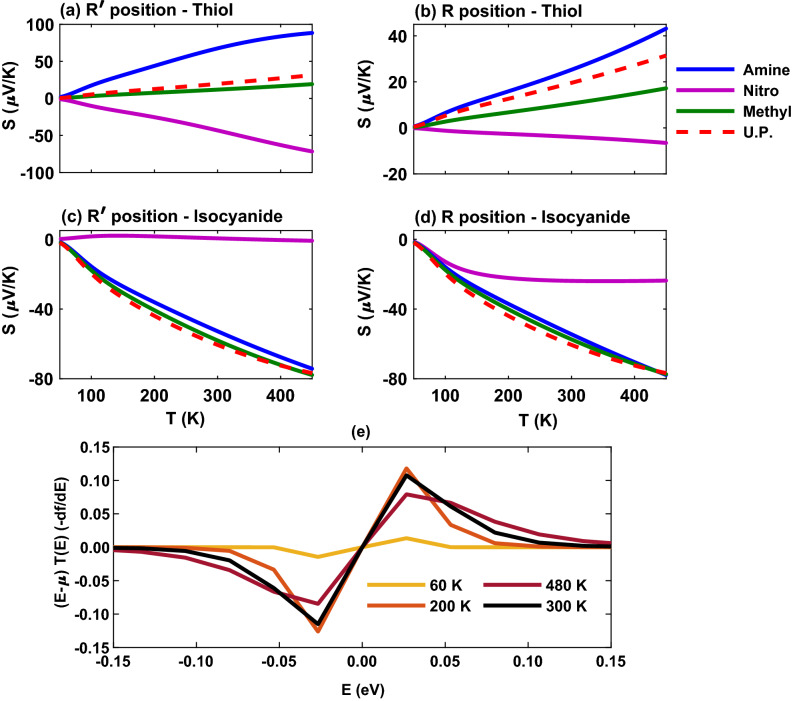
The thermopower of the Au-anthracene-Au junction versus temperature when the molecule-electrode contact in mediated by the thiol anchoring unit in the presence of amine (blue), nitro (magenta) or methyl (green) as the side group in (**a**) $\mathrm{R}^{\prime }$ and (**b**) $\mathrm{R}$ positions. The electrical conductance of the isocyanide-terminated junction with amine (blue), nitro (magenta) or methyl (green) side group in (**c**) $\mathrm{R}^{\prime }$ and (**d**) $\mathrm{R}$ positions. Also, the thermopower of the unperturbed (U.P.; red) system in the absence of side groups is drawn as a reference. (**e**) $(\varepsilon -\mu ) T(\varepsilon )(-df/d\varepsilon )$ is drawn for the isocyanide-terminated junction with nitro side group in the $\mathrm{R}^{\prime }$ position.

Among thermoelectric coefficients, the role thermopower in shaping the behavior of the figure of merit may be more critical since the figure of merit depends on the square of the thermopower. Moreover, the sign of thermopower can determine the conductance mechanism such that the negative (positive) sign indicates that electrons (holes) are responsible for the charge transfer in the molecular junction. The location of FMOs with respect to the Fermi energy level can also be found from the sign of thermopower so that negative (positive) sign indicates a LUMO-dominated (HOMO-dominated) charge transfer. Therefore, the sign of the thermopower is mainly determined by the charge donating/accepting character of the anchoring groups^34^. The electron-donating thiol unit leads to a positive thermopower whereas the electron-accepting isocyanide anchoring group results in a negative thermopower. Furthermore, this can be inferred from the electronegativity of the anchoring group and that of the contacting Au electrodes, as argued earlier.

The thermopower of the molecular junction with side groups is shown in Fig. 9a–d. In the thiol-terminated molecule with electron-donating amine (blue) and methyl (green) side groups the thermopower is positive and increases with temperature in both $\mathrm{R}^{\prime }$ and $\mathrm{R}$ positions (Fig. 9a and b). However, the molecule with electron-accepting nitro side group overcomes the charge transfer nature of the anchoring unit and leads to a negative thermopower that is decreasing with temperature (Fig. 9a and b, magenta). The magnitude of the thermopower, however, is determined by the location of HOMO or LUMO peak relative to the Fermi energy in the transmission spectrum. For example, as shown in Fig. 6a the transmission peaks of the thiol-terminated molecule with amine (blue) and nitro (magenta) side groups were located closer to the Fermi energy in comparison to the methyl (green) unit leading to greater thermopower in these cases in Fig. 9a in the $\mathrm{R}^{\prime }$ position. In the $\mathrm{R}$ position the thermopower is decreased for amine and nitro side groups in Fig. 9b but remained relatively unchanged for the methyl side group. This is due to the increased transmission $\mathrm{HOMO-LUMO}$ gap for amine and nitro units in the $\mathrm{R}$ position, except the methyl unit which was unaffected (see Fig. 6b). These observations can be further explained by the approximated expression $S \propto d\varepsilon \log (T(\varepsilon ))$ near the Fermi energy for the thermopower, i.e., the slope of the transmission is a function of energy on the logarithmic scale.

In the isocyanide-terminated molecule the maximum value of thermopower corresponds to the molecule with methyl (green) side group whereas the minimum value belongs to the nitro (magenta) unit in both $\mathrm{R}^{\prime }$ and $\mathrm{R}$ positions (Fig. 9c and d). This could be predicted by the peak of transmission spectrum of the molecule with methyl side group that is located near the Fermi energy in comparison to the other side groups (see Fig. 6c and d, green). The fluctuation of thermopower near zero for the molecule with nitro side group in the $\mathrm{R}^{\prime }$ position in Fig. 9c is due to the behavior of $(\varepsilon -\mu ) T(\varepsilon )(-df/d\varepsilon )$ shown in Fig. 9e that is associated with $L_1$ in the thermopower relation given by Eq. (5). In Fig. 9e, $L_1$ is increased up to $T \sim 200 \, \mathrm{K}$ around the Fermi energy and then decreased which is qualitatively appeared in the behavior of the thermopower for the molecule with nitro side unit in Fig. 9c. Furthermore, the small value of the thermopower in this case can be attributed to the nearly perfect symmetric shape of the transmission at the Fermi energy for the nitro-perturbed molecule (see Fig. 6c, magenta). In the $\mathrm R$ position, however, the value of thermopower for the nitro side group is increased (and shifted toward negative values), but in the case of amine (blue) and methyl (green) side groups the behavior of thermopower is relatively similar to the $\mathrm{R}^{\prime }$ position. This is due to the observation that the $\mathrm{HOMO-LUMO}$ gap for the nitro unit has been slightly shifted towards higher energies in the transmission spectrum in the $\mathrm{R}$ position (Fig. 6d), however, it remained relatively unchanged for amine and methyl side groups. Moreover, it is notable that in the $\mathrm R$ position of side groups in the isocyanide-terminated molecule the sign of the thermopower is negative irrespective of the charge transfer nature of the side group.

**Figure 10:**
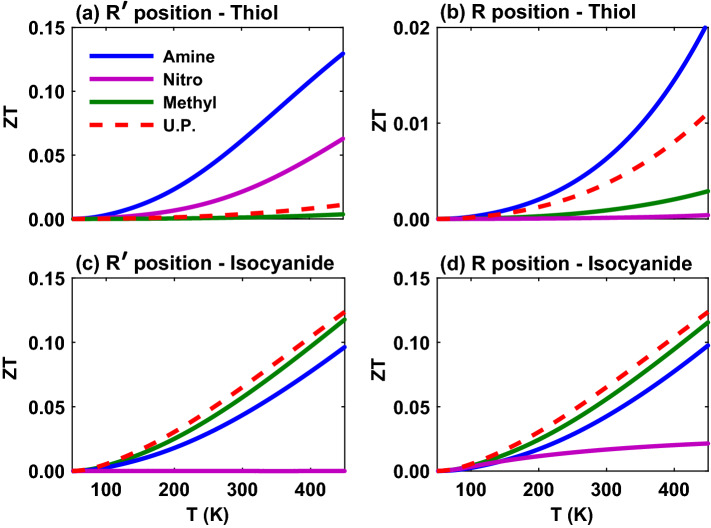
The figure of merit of the Au-anthracene-Au junction as a function of temperature for the thiol-terminated junction with amine (blue), nitro (magenta) or methyl (green) as the side group in (**a**) $\mathrm{R}^{\prime }$ and (**b**) $\mathrm{R}$ positions. The electrical conductance of the isocyanide-terminated junction with amine (blue), nitro (magenta) or methyl (green) side group in (**c**) $\mathrm{R}^{\prime }$ and (**d**) $\mathrm{R}$ positions. Furthermore, the figure of merit of the unperturbed (U.P.; red) system in the absence of side groups is drawn as a reference.

The interplay between electrical conductance, thermal conductance and thermopower determines the figure of merit of the system that is shown in Fig. 10 as a function of temperature. The thiol-terminated molecule with amine (blue) side group has the greatest figure of merit in both $\mathrm{R}^{\prime }$ and $\mathrm R$ positions (Fig. 10a and b) apparently due to the greater contributions from the electrical conductance (Fig. 7a and b) and thermopower (Fig. 9a and b). However, the minimum value of figure of merit in the $\mathrm{R}^{\prime }$ position corresponds to the methyl (green) side group whereas in the $\mathrm{R}$ position the nitro side group has the lowest figure of merit. These results can be explained by the behavior of their corresponding electrical conductance (Fig. 7a and b) and thermopower (Fig. 9a and b) that were lower than that of the other side groups. The main difference between the two positions of side groups is that the figure of merit for amine and nitro side groups is reduced in the $\mathrm{R}$ position but remained sort of unaffected for the methyl unit. In either case, the figure of merit has been improved with temperature.

In the isocyanide-terminated molecular junction, however, the molecule with methyl (green) side group shows the greatest figure of merit in both $\mathrm{R}^{\prime }$ and $\mathrm{R}$ positions (Fig. 10c and d) mostly due to its greater thermopower in magnitude (Fig. 9c and d). It is notable that although the electrical conductance for the nitro-perturbed molecule is higher than other side groups (see Fig. 7c and d, magenta), its suppressed thermopower (Fig. 9c and d) prevents nitro from reaching high values of the figure of merit (Fig. 10c and d) in this case, and therefore, the lowest figure of merit belongs to nitro side group in both positions. Furthermore, another notable observation is that the figure of merit of isocyanide-terminated molecular junction is fairly robust to conformational changes of side group position by shifting from $\mathrm{R}^{\prime }$ to $\mathrm{R}$.

Ultimately, in order to compare the effect of side group position and its charge transfer nature on the thermoelectric properties of the anthracene molecular junction with anchoring groups, the results were summarized in Table 1 which have been evaluated at $T = 300 \, \mathrm{K}$. For easier inspection, the chemical formula of each side group is color coded similar to those used in the depiction of thermoelectric coefficients in Figs. 7–10a–d. Table 1 shows that in the molecule with thiol anchoring group, at $T = 300 \, \mathrm{K}$ the behavior of all thermoelectric coefficients in the presence of side groups is similar, i.e. amine > nitro > methyl in the $\mathrm{R}^{\prime }$ position and amine > methyl > nitro in the $\mathrm{R}$ position. However, in the isocyanide-terminated molecular junction the behavior of thermoelectric coefficients is more complex. The trend of electrical conductance at $T = 300 \, \mathrm{K}$ is robust to the side group conformational change, i.e. nitro > methyl > amine in both positions. In the thermal conductance the trend of nitro and methyl side groups is replaced by changing the side group position from $\mathrm{R}^{\prime }$ (methyl > nitro > amine) to $\mathrm{R}$ (nitro > methyl > amine). However, the trend of thermopower and figure of merit is similar so that methyl > amine > nitro which is robust to the conformational changes of side groups at $T = 300 \, \mathrm{K}$.

**Table 1:** Comparative results for thermoelectric properties of the anthracene molecular junction with amine ($- \mathrm{NH}_2$), nitro ($- \mathrm{NO}_2$) or methyl ($- \mathrm{CH}_3$) as the side group in the $\mathrm{R}^{\prime }$ and $\mathrm{R}$ positions mediated by thiol ($- \mathrm{SH}$) or isocyanide ($- \mathrm{NC}$) as the anchoring group in the $\mathrm{X}$ position evaluated at $T = 300 \, \mathrm{K}$.

Coef.	Side group in the $\mathrm {R}^{\prime }$ position		Side group in the $\mathrm{R}$ position	
	$\mathrm{-SH}$ anchoring group	$\mathrm{-NC}$ anchoring group	$\mathrm{-SH}$ anchoring group	$\mathrm{-NC}$ anchoring group
*G*	${-\mathrm {NH}_2}> {-\mathrm {NO}_2} > {-\mathrm {CH}_3}$	${-\mathrm {NO}_2}> {-\mathrm {CH}_3} > {-\mathrm {NH}_2}$	${-\mathrm {NH}_2}> {-\mathrm {CH}_3} > {-\mathrm {NO}_2}$	${-\mathrm {NO}_2}> {-\mathrm {CH}_3} > {-\mathrm {NH}_2}$
$K_{\mathrm {el}}$	${-\mathrm {NH}_2}> {-\mathrm {NO}_2} > {-\mathrm {CH}_3}$	${-\mathrm {CH}_3}> {-\mathrm {NO}_2} > {-\mathrm {NH}_2}$	${-\mathrm {NH}_2}> {-\mathrm {CH}_3} > {-\mathrm {NO}_2}$	${-\mathrm {NO}_2}> {-\mathrm {CH}_3} > {-\mathrm {NH}_2}$
*S*	${-\mathrm {NH}_2}> {-\mathrm {NO}_2} > {-\mathrm {CH}_3}$	${-\mathrm {CH}_3}> {-\mathrm {NH}_2} > {-\mathrm {NO}_2}$	${-\mathrm {NH}_2}> {-\mathrm {CH}_3} > {-\mathrm {NO}_2}$	${-\mathrm {CH}_3}> {-\mathrm {NH}_2} > {-\mathrm {NO}_2}$
*ZT*	${-\mathrm {NH}_2}> {-\mathrm {NO}_2} > {-\mathrm {CH}_3}$	${-\mathrm {CH}_3}> {-\mathrm {NH}_2} > {-\mathrm {NO}_2}$	${-\mathrm {NH}_2}> {-\mathrm {CH}_3} > {-\mathrm {NO}_2}$	${-\mathrm {CH}_3}> {-\mathrm {NH}_2} > {-\mathrm {NO}_2}$

## Discussion

In this study, by employing DFT calculations combined with NEGF formalism in linear response regime we theoretically analyzed the thermoelectric properties of an anthracene single-molecule junction that is connected to Au electrodes via thiol ($- \mathrm{SH}$) or isocyanide ($- \mathrm{NC}$) anchoring group. Particularly, we were interested in how these thermoelectric properties are modified when the molecule is functionalized by different side groups in comparison to the unperturbed molecule (i.e. without side groups). We considered three side groups, i.e. amine ($- \mathrm{NH}_2$), nitro ($- \mathrm{NO}_2$) and methyl ($- \mathrm{CH}_3$), in two different positions indicated by $\mathrm{R}^{\prime }$ and $\mathrm{R}$ labels in Fig. 1a and explored the behavior of thermoelectric coefficients in various configurations. The results showed that the interplay between charge transfer character of side groups and anchoring group as well as the position of side groups ultimately determines thermoelectric properties of the system.

We considered both electron-donating (amine and methyl) and electron-accepting (nitro) side groups in two different positions for the functionalization of the molecule that its contact with the electrodes were mediated by electron-donating (thiol) and electron-accepting (isocyanide) anchoring groups to comparatively examine the effect of side group position and its charge transfer properties on thermoelectric coefficients. We found that when the molecule was anchored with the electron-donating thiol unit, in the case of electron-donating amine and methyl side groups the transmission was HOMO-dominated whereas the electron-accepting nitro side group led to a LUMO-dominated transmission in both $\mathrm{R}^{\prime }$ and $\mathrm{R}$ positions (Fig. 6a and b). However, it was notable that in the molecule terminated with the electron-accepting isocyanide unit the transmission was LUMO-dominated for all of the side groups irrespective of their position or their charge transfer nature in both positions (Fig. 6c and d). In addition, the main different between $\mathrm{R}^{\prime }$ and $\mathrm{R}$ positions of side groups was that the $\mathrm{HOMO-LUMO}$ gap in the transmission spectrum was relatively increased in the $\mathrm{R}$ position.

Alternation in the position or the chemical nature of side groups can lead to substantial variations of the transmission coefficient, mostly due to changes in the alignment of the FMOs relative to the Fermi energy^40,41^ that can ultimately modulate figure of merit and thermopower^5^. This was previously addressed by a number of studies which revealed that charge transport polarization in the molecule can be controlled either by chemically modifying the side group or by changing the conformation of the side group^4,39–41,47^. Furthermore, previous studies showed that the position or the charge transfer nature of anchoring groups may play a critical role in the determination of thermoelectric properties (e.g. the thermopower and figure of merit) in molecular devices by modifying the shape or location of peaks in the transmission spectrum^34,46^.

We assumed two positions for side groups shown by $\mathrm{R}^{\prime }$ and $\mathrm{R}$ in Fig. 1a. However, similar studies investigated the effect of other spatial configurations^47^ or rotational variations of the side group relative to the molecular backbone^4,41^ on the thermopower and figure of merit in single-molecule devices. Moreover, here the contact of the molecule with the electrodes was mediated by anchoring groups only in the $\mathrm{X}$ position shown in Fig. 1a. Previous studies, however, showed different positions of anchoring groups can have modulatory effects on the transport properties of single-molecule junction by changing the $\mathrm{HOMO-LUMO}$ symmetry in the spatial distribution of the molecular orbitals that leads to symmetry-allowed or symmetry-forbidden electron transport through the system^33,66^. These conformational-induced modifications of the transport properties of molecular junctions in the presence of side groups or anchoring groups as well as the chemical nature of these groups^34,46^ can directly or indirectly impact on the thermoelectric properties on the system.

Our results showed that in the thiol-terminated molecule the trend of electrical conductance, thermal conductance, thermopower and figure of merit is similar in each side group position, e.g. at $T = 300 \, \mathrm{K}$ (Table 1): amine > nitro > methyl in the $\mathrm{R}^{\prime }$ position and amine > methyl > nitro in the $\mathrm{R}$ position. In the isocyanide-terminated system, however, the thermopower and figure of merit followed each other’s trend so that methyl > amine > nitro in both $\mathrm{R}^{\prime }$ and $\mathrm{R}$ positions. Generally, the thermoelectric coefficients were more robust to the change of side group position in the isocyanide-terminated molecule. Particularly, independent of the side group position, the molecule with amine side group showed the greatest value of figure of merit when thiol was the anchoring unit (see Fig. 10a and b), whereas in the isocyanide-terminated molecule the system with methyl side group attained the the greatest value of figure of merit (see Fig. 10c and d). Moreover, the comparison between the molecule terminated with thiol and isocyanide units shows that the magnitude of figure of merit in the isocyanide-terminated molecule is fairly robust to the side group position, however, the figure of merit of the thiol-terminated molecule in the $\mathrm{R}$ position is generally reduced relative to the $\mathrm{R}^{\prime }$ position.

As shown in Figs. 7a–d and 8a–d the magnitude of electrical and thermal conductances for the thiol-terminated molecule perturbed with side groups is smaller than the isocyanide-terminated molecular junction in both $\mathrm{R}^{\prime }$ and $\mathrm{R}$ position. The greater electrical and thermal conductances in the isocyanide-terminated molecule may be due to the binding strength of the isocyanide to the Au surfaces that is weaker than that of the thiol unit; hence, the isocyanide group can be adsorbed onto the Au surfaces^67^. This ultimately enhances the transmission leading to greater conductance for the isocyanide anchoring group relative to the thiol unit^68^. Furthermore, it is notable that when the anchoring group is electron-donating (i.e. thiol), $\mathrm{R}^{\prime }$ position of the electron-accepting nitro side group enhances the magnitude of thermopower and thermoelectric efficiency (i.e. the figure of merit) in Figs. 9a and 10a. However, when the anchoring group is electron-accepting (i.e. isocyanide) the figure of merit is suppressed in the case of nitro side group in the $\mathrm{R}^{\prime }$ position (Figs. 9c and 10c). This is due to the fact that the nitro side group is considerably more electronegative than the thiol anchoring group leading to the greater donation of electron density into the Au surface in the $\mathrm{R}^{\prime }$ position that provides more stabilization of the $\mathrm{Au-SH}$ bond^69^ which in turn increases the thermoelectric efficiency.

Similar previous studies have shown that thermal conductance in single-molecule junctions with Au electrodes where the central molecule is a polycyclic organic compound is significantly dominated by electrons^18,38,70^, such that the maximum of phonon contribution to the thermal conductance as a function of temperature saturates to $\sim 25 \, \mathrm{pW/K}$ in theoretical^38^ or to $\sim 20 \, \mathrm{pW/K}$ in recent experimental studies^18^. Therefore, the phonon contribution to the thermal conductance is typically ignored in thermoelectric effect studies in such molecular junctions due to the low Debye frequency of the Au electrodes compared to the frequency of molecular vibrational modes that substantially restricts phonon transport in the system^18^. Here, in accordance with these findings, we reasonably assumed a constant value of $30 \, \mathrm{pW/K}$ for the phononic thermal conductance to provide a more realistic representation for the figure of merit^18,38^. A more accurate estimation of the figure of merit, however, requires careful inspection of the phonon contribution to the thermal conductance that remains open for the future studies.

The Wiedemann–Franz law is predicted to be valid for certain metals, but when it comes to the nanoscale devices its validity is under debate. Recently, novel experimental techniques were developed to precisely measure the thermal and electrical transport in certain atomic-sized contacts^71,72^, verifying the validity of the Wiedemann–Franz law at this scale. However, rigorous theoretical studies questioned the generality of these observations and argued that notable deviations can be seen depending on the molecular structure, temperature or gate voltage (e.g. the Lorenz number may increase up to $\sim 30\%$ for Au)^73^. Since both the electrical conductance and electronic thermal conductance were calculated here, we quantitatively checked if the Wiedemann-Franz law holds in the Au-anthracene-Au single-molecule junction considered in our study. The calculated Lorenz ratios (i.e. ${\mathcal {L}}/{\mathcal {L}}_0$) defined in Eq. (S1) in the Supplementary Information are plotted in Fig. S1 as a function of temperature for both the unperturbed molecule (i.e. without side group) and perturbed molecule (i.e. with side groups in the $\mathrm{R}^{\prime }$ and $\mathrm{R}$ positions) anchored with the thiol or isocyanide unit. Figure S1 shows deviations from the Wiedemann–Franz law in the Au-anthracene-Au molecular junction considered in our study, irrespective of the presence, chemical nature or position of side groups for both anchoring groups. It has been suggested that phonon contributions may cause deviations from the Wiedemann–Franz law in metallic atomic-size contacts^70,74^, however, quantum effects and energy dependency of the electronic transmission near the Fermi energy can lead to significant violation of the Wiedemann–Franz law in molecular junctions^73^ as it is the case here. Particularly, as illustrated in Figs. 2 and 6, non-smooth dependence of the transmission spectrum to energy leads to a relatively monotonic increase of the electronic thermal conductance with temperature (as argued in the discussion of Fig. 8a–d) compared to the trend of the electrical conductance that is less temperature-dependent (as argued in the discussion of Fig. 7a–d). This different modulation of electronic thermal conductance and electrical conductance ultimately increased the calculated Lorenz ratio leading to deviations from the Wiedemann-Franz law (see Fig. S1).

Ultimately, the side groups in the perturbed molecule can act as a chemical gate that shift the peaks of the transmission spectrum relative to the Fermi energy in comparison to the unperturbed molecule. The interplay between the chemistry of the side groups and anchoring groups shapes the nature of electronic transport and the resultant thermopower and figure of merit. Our comparative results may be of interest of theoretical and experimental studies for an appropriate choice of functionals in order to obtain higher values of thermoelectric efficiency in single-molecule junctions. This can finally lead to the design and fabrication of state-of-the-art molecular junctions with desirable thermoelectric features that certainly deserves more theoretical, computational and experimental scrutiny.

## Supplementary Information

Supplementary Information 1.

## Data Availability

All data generated or analysed during this study are included in this published article and its supplementary information files.
