# Supplementary material for: Side-group-mediated thermoelectric properties of anthracene single-molecule junction with anchoring groups
Source: Sci Rep. 2021 Apr 26;11:8958. doi: 10.1038/s41598-021-88297-2 (PMC8076224; doi:10.1038/s41598-021-88297-2)
Supplement: Supplementary file 1 — Supplementary Information 1. [file 41598_2021_88297_MOESM1_ESM.pdf]

Supplementary Information for: Side-group-mediated thermoelectric properties of anthracene single-molecule junction with anchoring groups

S. Ramezani Akbarabadi[1,*], H. Rahimpour Soleimani[1], and M. Bagheri Tagani[1]

[1]**Computational Nanophysics Laboratory (CNL), Department of Physics, University of Guilan, Rasht, 41335-1914, Iran**

[*]**sramezani@phd.guilan.ac.ir**

Violation of the Wiedemann-Franz law

In order to quantitatively check the validity of the Wiedemann-Franz law in the Au-anthracene-Au single-molecule junction in our study, we defined the Lorenz ratio as follows [1]:

$$\frac{\mathcal{L}}{\mathcal{L}_0} = \frac{K_{\text{el}}}{\mathcal{L}_0 T G}, \tag{S1}$$

where $\mathcal{L}$ is the Lorenz number that reaches the Sommerfeld value, i.e. $\mathcal{L}_0 = \pi^2 k_{\text{B}}^2/(3e^2) = 2.44 \times 10^{-8}\ \text{W/SK}^2$, when the Wiedemann-Franz law holds (i.e. $\mathcal{L}/\mathcal{L}_0 = 1$). We calculated the Lorenz ratio as a function of temperature for both the unperturbed molecule (i.e. without side group) and perturbed molecule (i.e. with side groups in the R′ and R positions) anchored with the thiol or isocyanide unit. Figure **S1** shows that the Wiedemann-Franz law is violated in the Au-anthracene-Au molecular junction considered in our study, irrespective of the presence, chemical nature or position of side groups for both anchoring groups.

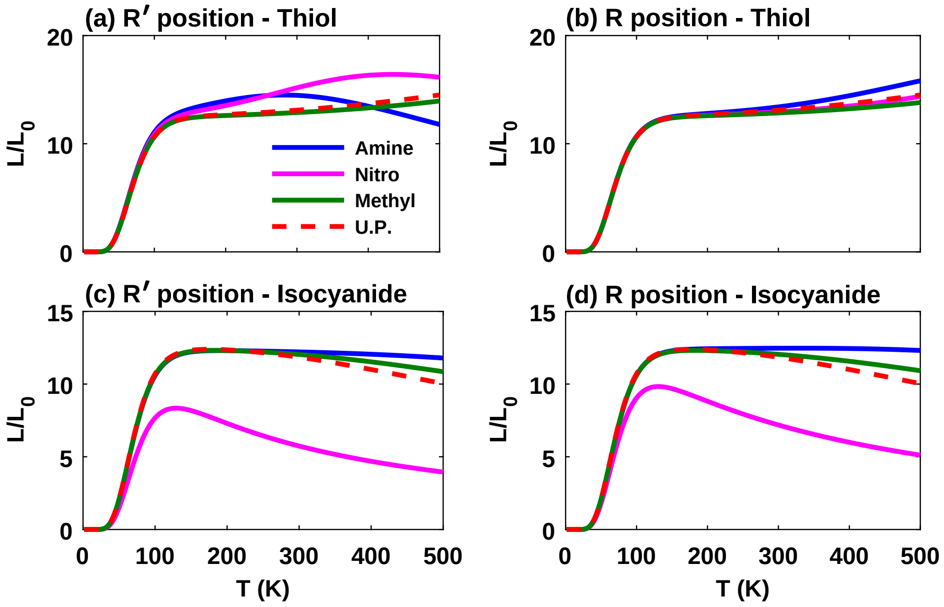

Figure S1. Lorenz ratios defined by Eq. (S1) as a function of temperature for unperturbed/perturbed molecule with the thiol anchoring group in the (**a**) R′ and (**b**) R positions. The same Lorenz ratio is drawn when the molecule is anchored with the isocyanide anchoring group in the (**c**) R′ and (**d**) R positions.

References

[1] Klöckner, J. C., Matt, M., Nielaba, P., Pauly, F. & Cuevas, J. C. Thermal conductance of metallic atomic-size contacts: phonon transport and Wiedemann-Franz law. *Phys. Rev. B* **96**, 205405 (2017).
